# Assessment of associations between neutrophil extracellular trap biomarkers in blood and thrombi in acute ischemic stroke patients

**DOI:** 10.1007/s11239-024-03004-y

**Published:** 2024-06-09

**Authors:** Tristan Baumann, Nicole de Buhr, Nicole Blume, Maria M. Gabriel, Johanna Ernst, Leonie Fingerhut, Rabea Imker, Omar Abu-Fares, Mark Kühnel, Danny D. Jonigk, Friedrich Götz, Christine Falk, Karin Weissenborn, Gerrit M. Grosse, Ramona Schuppner

**Affiliations:** 1 Department of Neurology, Hannover Medical School, Carl-Neuberg-Str. 1, 30625 Hannover, Germany; 2 Institute of Biochemistry, University of Veterinary Medicine Hannover, Hannover, Germany; 3 Research Center for Emerging Infections and Zoonoses (RIZ), University of Veterinary Medicine Hannover, Hannover, Germany; 4 Institute of Diagnostic and Interventional Neuroradiology, Hannover Medical School, Hannover, Germany; 5 Institute of Pathology, Hannover Medical School, Hannover, Germany; 6 Member of the German Center for Lung Research (DZL), Biomedical Research in Endstage and Obstructive Lung Disease Hannover (BREATH), Hannover, Germany; 7 Institute of Pathology, RWTH Aachen Medical University, Aachen, Germany; 8 Institute of Transplant Immunology, Hannover Medical School, Hannover, Germany; 9 Department of Neurology and Stroke Center, University Hospital Basel, Basel, Switzerland

**Keywords:** Stroke, Neutrophil extracellular traps, NETs, Thrombo-inflammation, Biomarkers

## Abstract

Inflammation including immunothrombosis by neutrophil extracellular traps (NETs) has important implications in acute ischemic stroke and can affect reperfusion status, susceptibility to stroke associated infections (SAI) as well as functional clinical outcome. NETs were shown to be prevalent in stroke thrombi and NET associated markers were found in stroke patients’ blood. However, little is known whether blood derived NET markers reflect the amount of NETs in thrombi. Conclusions from blood derived markers to thrombus composition might open avenues for novel strategies in diagnostic and therapeutic approaches. We prospectively recruited 166 patients with acute ischemic stroke undergoing mechanical thrombectomy between March 2018 and May 2021. Available thrombi (n = 106) were stained for NET markers DNA-histone-1 complexes and myeloperoxidase (MPO). Cell free DNA (cfDNA), deoxyribonuclease (DNase) activity, MPO-histone complexes and a cytokine-panel were measured before thrombectomy and after seven days. Clinical data, including stroke etiology, reperfusion status, SAI and functional outcome after rehabilitation, were collected of all patients. NET markers were present in all thrombi. At onset the median concentration of cfDNA in blood was 0.19 µg/ml increasing to 0.30 µg/ml at 7 days. Median DNase activity at onset was 4.33 pmol/min/ml increasing to 4.96 pmol/min/ml at 7 days. Within thrombi DNA-histone-1 complexes and MPO correlated with each other (*ρ* = 0.792; *p* < 0.001). Moreover, our study provides evidence for an association between the amount of NETs and endogenous DNase activity in blood with amounts of NETs in cerebral thrombi. However, these associations need to be confirmed in larger cohorts, to investigate the potential clinical implications for individualized therapeutic and diagnostic approaches in acute ischemic stroke.

**Graphical Abstract:**

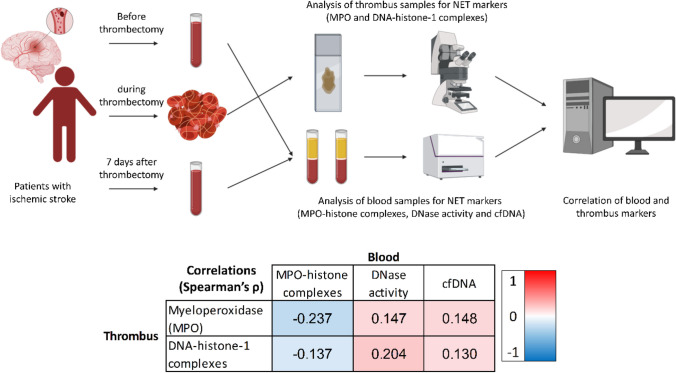

**Supplementary Information:**

The online version contains supplementary material available at 10.1007/s11239-024-03004-y.

## Introduction

Inflammatory mechanisms are a hallmark in the pathophysiology of ischemic stroke [1, 2]. Systemic inflammation can trigger immuno-thrombosis, e.g. by formation of neutrophil extracellular traps (NETs), which have already been found in cerebral thrombi of stroke patients [3]. NETs have been first described in 2004 [4]. Neutrophil granulocytes have the ability to release their decondensed chromatin as the backbone of NETs, and granular proteins that are bound to this structure. These NETs can then entrap and kill microorganisms, for which they contain high levels of antimicrobial substances [4] as part of the innate immunity. However, detrimental effects have been shown as well: NETs promote the aggregation of platelets through various mechanisms, such as the direct activation of platelets by histones or indirectly by binding of prothrombotic substances like von Willebrand factor (vWF) [5]. NETs have been shown to have important implications for several clinical stroke characteristics and outcomes such as reperfusion during mechanical thrombectomy or thrombolysis [6, 7] and stroke etiology [8]. Less, however, is known on whether markers of NETs in the circulation may reflect the actual prevalence of NETs within cerebral thrombi. If blood and thrombus content were found to be associated, this could potentially provide diagnostic benefits and also unveil new therapeutic opportunities.

Recent studies emphasized already the clinical relevance of NETs in stroke patients by showing that higher concentrations of NETs in thrombi result in longer and less successful mechanical thrombectomies (MT) [6, 7]. The application of deoxyribonuclease I (DNase I) was shown to contribute to lysis of NET-rich thrombi ex vivo [3, 6]. Additionally, the amount of DNA in thrombi seems to differ for different origins of stroke [8], underlining the potential relevance in secondary preventive considerations. However, as not all mechanical thrombectomies are successful to achieve reperfusion [9], surrogate blood markers might present possibilities for further diagnosis.

Several NET markers are described that can be measured in blood, like citrullinated histones, cell free DNA (cfDNA), nucleosomes and MPO-DNA complexes. All of them were detected in blood of patients with ischemic stroke [10, 11]. These markers were also shown to be associated with stroke origin, severity and clinical outcome [10, 12]. For example, cfDNA was found to be a predictor for clinical outcome, especially mortality, and increase in cfDNA over time was associated with stroke associated infections (SAI)[13]. Recently the efficient degradation of cfDNA via DNase was proposed to reduce post-stroke lymphocytopenia, which in turn is known to be associated with increased susceptibility for SAI [14]. Additionally, concentrations of cfDNA also correlated with specific cytokines, namely Interleukin (IL)-1 β, IL-1 receptor antagonist (RA), IL-2, IL-6, tumor necrosis factor (TNF)-α, IL-10, interferon (IFN)-α, macrophage inflammatory protein (MIP)-1β and IL-17F [13].

However, prior studies focused either on NETs in thrombi [3, 6, 7] or circulating NET markers in blood [10, 11]. Knowledge about the correlation of these circulating NET markers in blood and the presence of NETs in cerebral thrombi is limited. In case of an association between markers of inflammation and especially NET markers in blood and in the cerebral thrombus, conclusions might be drawn from a peripheral blood sample to thrombus composition. Furthermore, nothing is known about the influence of regulatory mechanisms like endogenous DNase activity on the composition of the thrombi. As a consequence, individualized therapeutic approaches, e.g. the use of NET degrading DNase I [3, 6], are conceivable.

Therefore, in this study we investigated the correlation between NET markers in blood samples as well as DNase activity, taken in the hyper-acute setting of stroke and one week after, and the amount of NETs in thrombi retrieved from patients with acute ischemic stroke. We also assessed the association of NETs in thrombi and circulating NET markers with clinical outcome parameters i.e. recanalization, stroke associated infections (SAI), functional outcome and etiology. Additionally, we analyzed an array of cytokines to confirm correlations between cytokines and NET markers found earlier [13] and to explore further possible links between NETs in thrombi and systemic cytokine reactions.

## Materials and methods

We prospectively recruited 166 patients who underwent MT for acute ischemic stroke with large vessel occlusion (LVO) at Hannover Medical School during two time periods between March 2018 and August 2019 (n = 92) (cohort 1) and between June 2020 and May 2021 (n = 74) (cohort 2). Additional criteria required for inclusion was the successful retrieval of a blood sample prior to MT. In cohort 1, samples were taken before thrombectomy by a venous blood sampling. Samples of cohort 1 were acquired during a former study by our group, as reported previously [13]. In cohort 2, blood was taken via the arterial groin puncture used for thrombectomy, immediately before the procedure. This was implemented to further streamline study procedures. We excluded all patients with known malignoma, immunodeficiency or ongoing medication with immunomodulatory drugs. Stroke thrombi were retrieved during MT. After seven days, venous blood samples were collected for both cohorts. An overview of the recruitment process, as well as available material for the different analyses, can be found in supplemental figure SFig. 1.

### Clinical data

Clinical data were gathered at inclusion and seven days after initial treatment, using a standardized electronic case report form. The collected data include demographic data, basic clinical parameters, vascular risk factors evaluated via the Essen Stroke Risk Score (ESRS) [15] and stroke severity at baseline determined by the National Institutes of Health Stroke Scale (NIHSS) obtained in the emergency department. Additionally, the 7 day follow-up included the occurrence of stroke associated infections (SAI) according to Centers for Disease Control and Prevention’s (CDC) Criteria [16]. The SAI status of patients who received antibiotic therapy without meeting the CDC criteria was labeled as indeterminate. Cerebral reperfusion was evaluated according to the modified Treatment in Cerebral Infarction (mTICI) score, graded by board certified neuroradiologists. An mTICI score of 2c or 3 in the anterior circulation or of 2b or higher in the posterior circulation was considered as sufficient reperfusion [17]. Both status of infection and mTICI were rated by two observers separately, before systematically agreeing on a consensus. The investigators grading the status of infection and the mTICI score were blinded to the patients’ biomarker data. Thrombus origin was classified at discharge according to the Trial of Org 10172 in Acute Stroke Treatment (TOAST) criteria [18].

Finally, for cohort 2 we scored the patients’ outcome with the modified Rankin Scale (mRS) [19] after the end of rehabilitation, based on the documentation of the respective inpatient rehabilitation centers. A mRS score of 2 or lower was considered as a favorable functional outcome.

### Analysis of thrombus composition

The stroke thrombi were analyzed in batches of 10 thrombi at a time. After retrieval, the thrombi were immediately fixed in 4% buffered formalin and later embedded in paraffin. For staining, 2 µm thick sections were cut from each thrombus and subjected to immunofluorescence staining as described previously [20]. Briefly, NETs were stained using as primary antibodies: a mouse monoclonal antibody (IgG2a) against DNA/histone 1 (MAB3864; Sigma Aldrich, Millipore 0.55 mg/mL diluted 1:100, Billerica, MA, USA) and a rabbit antibody (IgG) against anti-human myeloperoxidase (A039829-2 Agilent, Santa Clara, CA, USA, 3.3 mg, 1:300). These were incubated overnight at 4 °C. The respective isotype control was included in each staining batch of analyzed thrombi. Therefore, murine IgG2a (from murine myeloma, M5409-1 mg conc. 0.2 mg/mL, 1:36,4 Sigma Aldrich, Munich, Germany) and rabbit IgG (from rabbit serum, Sigma Aldrich, Munich, Germany, I5006, 1.16 mg/mL, 1:96,7) was incubated under the same conditions as the primary antibodies.

As secondary antibodies goat anti-mouse Alexa 488Plus 2 mg/ml (# A32723, Invitrogen, Carlsbad, CA, USA) and goat anti-rabbit Alexa 633 2 mg/ml (# A21070, Invitrogen, Carlsbad, CA, USA, 2 mg, Waltham, MA, USA), both diluted 1:500 in blocking buffer, were used. Finally, all samples were processed using the TrueVIEW autofluorescence quenching kit (# SP-8400–15, Vector Laboratories, Newark, California, United States) following the manufacturer’s instructions and counterstained using Hoechst 33,342 (1:1000, stock 50 mg/mL, Sigma Aldrich, Munich, Germany).

**Fig. 1:**
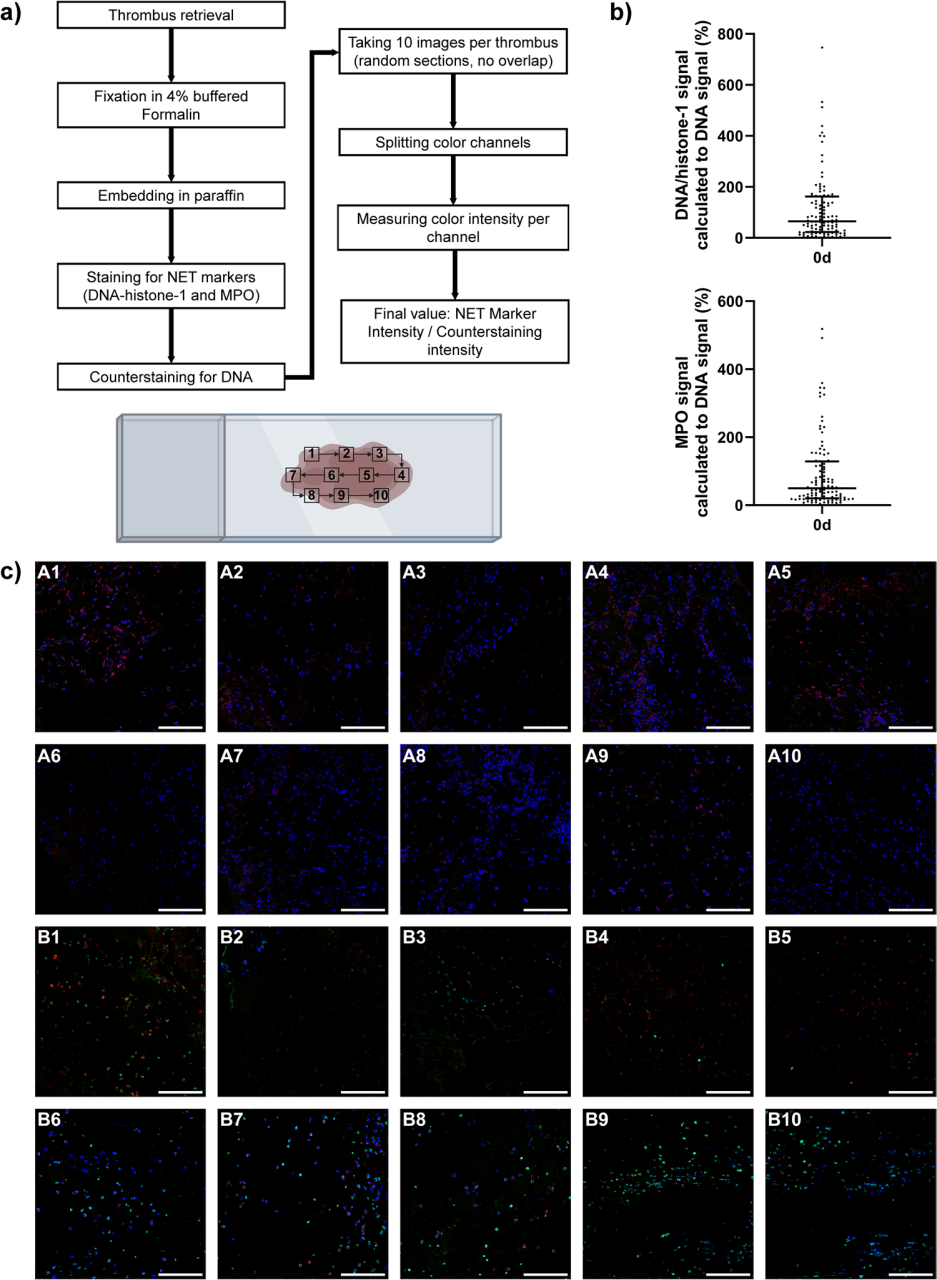
**a** The figure contains a flowchart, describing the process of NET marker measurement in the thrombi. 10 images were taken randomly and without overlap from the entirety of the thrombus. The pattern used to ensure no overlap can be seen below. After imaging the color channels were split and their intensity measured separately. The final value was then determined by dividing the NET marker intensity by the DNA counterstaining. **b** Scatterplots of the analyzed thrombus markers DNA-histone 1 complexes (green) and MPO (red) are presented. The integrated density in both channels as well as for the DNA-counterstaining (blue) was measured up to 10 pictures of each thrombus. The ratio was calculated and is presented as percentage. Each dot presents the mean of each individual patient. The bars show median and interquartile range. **c** 10 analyzed images of two thrombi (green: DNA-histone 1 complexes, red: MPO, blue: DNA counterstaining, bar length: 100 µm) are presented to show the distribution of NET markers in the thrombus. Image series A is from a thrombus with relative intensities in the first (DNA-histone 1 complexes) and second (MPO) quartile, while image series B shows a thrombus with relative intensities in the fourth quartile (both markers). Created with biorender.com, Microsoft Powerpoint, Adobe Illustrator, Adobe Photoshop and Image J

The samples were imaged using a Leica TCS SP5 AOBS confocal inverted-base fluorescence microscope with an HCX PL APO × 40 0.75–1.25 oil immersion objective. The settings for each batch were adjusted to their respective isotype controls. Up to 10 images were taken per thrombus, if the size of the thrombus was sufficient. If the thrombus was too small, the maximum amount of images possible was taken instead. The imaged areas were chosen randomly without overlap, from the entirety of the stained sample. The raw integrated density was then separately measured using ImageJ software (ver. 1.53e) for the three different color channels (blue = DNA [counterstaining], green = DNA/histone-1-complexes and red = MPO). The raw integrated density of both antibody signals was then set in relation to the raw integrated density of the counterstaining, to adjust for thrombus cell count. This ratio of signal intensity is therefore represented as percentage. Every batch was imaged within 24 h after the completion of the staining process. An overview of the thrombus analysis process, including the pattern used to select the imaged areas, can be seen in Fig. 1.

### MPO-histone complexes in blood

Myeloperoxidase (MPO)-histone complexes were measured using a previously established sandwich enzyme-linked immunosorbent assay (ELISA), as reported earlier by de Buhr et. al. [21]. For this we used components of the Cell Death Detection ELISAPLUS Kit (Roche11774425001). The additional antibodies used were a rabbit anti-human MPO antibody (Merck Millipore #07–496-I, 1 mg/mL; 1:200 diluted in 1% PBS-BSA (bovine serum albumin, Roth CP84.2 or 1ETA.2)) and goat anti-rabbit IgG HRP conjugated (Merck Millipore #12–348, 1:5000 diluted in PBS). For this analysis we used 100 µl of EDTA buffered plasma per sample at baseline and after seven days.

### Cell-free DNA in blood

Cell-free DNA was measured in 25 µl citrate buffered plasma at baseline and after seven days using a Quant-iT™ PicoGreen® assay (Invitrogen, Carlsbad, California, USA, P11496) as described previously [21].

### DNase activity in blood

DNase activity was measured in 25 µl serum at baseline and after seven days using a DNase I Activity Assay Kit (BioVision, Milpitas, California, USA, Fluorometric, K429-100), according to the manufacturer’s instructions.

### Cytokine analysis

The cytokine analysis was only conducted for cohort 2 since this has already been reported for cohort 1 in a previous work by our group [22]. The second group was analyzed using a MERCK Milliplex® Immunology Multiplex Assay (Merck Millipore #HCYTA-60 K-38C) according to the manufacturer’ s instructions. We analyzed EDTA buffered blood plasma samples taken at both baseline and after seven days.

### Statistical analysis

Statistical analyses were conducted using IBM SPSS Statistics Version 28 and 29. The correlation between thrombus composition and NET markers in blood was evaluated using the Spearman rank test. The same test was used to identify correlations between the various NET markers and cytokines. To evaluate potential differences in blood markers due to different approaches of sample collection (i.e., venous vs. arterial puncture), in both cohorts’ blood markers were compared using the Mann–Whitney-U-test. An evaluation of the NET markers and the clinical parameters and outcomes was conducted using Mann–Whitney-U-test and Kruskal–Wallis-H-test for continuous data. For categorical data the Chi square test or Fisher’s exact test were used, as appropriate. Binary logistic regression analysis was performed on the association of MPO-histone complexes and mTICI score adjusting for intravenous thrombolysis and stroke etiology. These confounders were identified by testing for significant differences between outcome groups using the above-mentioned statistical tests and were also theoretically reasoned confounders in the causal path between exposure and outcome.

## Results

### Clinical and demographic characteristics

The clinical characteristics and demographic data of all patients are presented in Table 1. No major differences between both patient cohorts could be found. Stroke severity at baseline was the same in both cohorts (NIHSS = 15). Both cohorts had a similar vascular risk profile before the event (ESRS = 3). Cohort 2 had a slightly higher mRS-Score before onset than cohort 1 (median 1 and 0, respectively). Also, the admission and treatment of patients, as measured by the onset to groin time was somewhat faster in cohort 2 (median 201 min and 253 min respectively). Patients in both cohorts primarily suffered from strokes in the anterior circulation (150 patients out of 166). In the majority of patients, the origin of stroke was classified as cardioembolic (n = 97; 58%), followed by strokes of cryptogenic origin (n = 42; 25%), large artery atherosclerosis (n = 24; 14%) and artery dissection (n = 3; 2%).

**Table 1:** Patient characteristics at baseline for all patients compared to the respective cohorts separately

Parameter	Type	All patients (n = 166)	Cohort 1 (n = 92)	Cohort 2 (n = 74)	*P*-value
Age, y	median (25th—75th percentile)	78 (67–83)	75.5 (64–82)	79.5 (69.75–84.25)	0.04
ESRS	median (25th—75th percentile)	3 (2–5)	3 (2–5)	3 (2–4)	0.52
NIHSS	median (25th—75th percentile)	15 (11–19)	15 (11–19)	15 (11–19)	0.66
Onset to groin time (when applicable), min	median (25th—75th percentile)	225.5 (148.25–311.25)	253 (159–256)	201 (136–266.25)	0.04
Door to groin time (when applicable), min	median (25th—75th percentile)	61 (38–86)	67 (39.5–90)	58 (36–85.25)	0.21
Onset to needle time (when applicable), min	median (25th—75th percentile)	91 (56–129.5)	115 (75–214.75)	93 (65–126)	0.12
mRS prior to stroke	median (25th—75th percentile)	0 (0–1)	0 (0–1)	1 (0–2)	< 0.01
Secondarily transferred to our hospital	n (%)	89 (53)	50 (54)	39 (52)	0.88
Sex (female)	n (%)	86 (52)	49 (53)	37 (50)	0.76
Arterial hypertension	n (%)	135 (81)	71 (77)	64 (87)	0.16
Diabetes	n (%)	51 (31)	28 (30)	23 (31)	0.93
Dyslipidemia	n (%)	61 (37)	30 (33)	31 (42)	0.26
Obesity (BMI > 30)	n (%)	44 (26)	27 (29)	17 (23)	0.38
Coronary heart disease	n (%)	31 (19)	22 (24)	9 (12)	0.07
Previous myocardial infarction	n (%)	21 (13)	15 (16)	6 (8)	0.16
Previous stroke or TIA	n (%)	32 (19)	16 (17)	16 (22)	0.56
Atrial fibrillation	n (%)	67 (40)	31 (34)	36 (49)	0.06
Intravenous thrombolysis	n (%)	101 (61)	55 (60)	46(62)	0.87
Unknown onset	n (%)	62 (37)	36 (39)	26 (35)	0.63
Smoker	n (%)	48 (29)	31 (34)	17 (23)	0.17
Alcohol addiction	n (%)	14 (8)	8 (9)	6 (8)	0.99
Affected territory of circulation					0.43
Anterior	n (%)	150 (90)	85 (92)	65 (88)	
ICA	n (%)	24 (14)	14 (15)	10 (14)	
MCA	n (%)	101 (61)	58 (63)	43 (58)	
ICA + MCA	n (%)	25 (15)	13 (14)	12 (16)	
Posterior	n (%)	16 (10)	7 (8)	9 (12)	
Etiology					0.24
Large artery atherosclerosis	n (%)	24 (14)	10 (11)	14 (19)	
Cardioembolic stroke	n (%)	97 (58)	54 (59)	43 (58)	
Cryptogenic stroke	n (%)	42 (25)	25 (27)	17 (23)	
Artery dissection	n (%)	3 (2)	3 (3)	0 (0)	

ESRS = Essen stroke risk score, NIHSS = National Institutes of Health Stroke Scale, mRS = modified Rankin Scale, BMI = body mass index, TIA = transient ischemic attack, ICA = internal carotid artery, MCA = middle cerebral artery

As for recanalization, in 82 (49%) patients a favorable reperfusion was achieved. 55 (33%) patients suffered from SAI, while 79 (47%) developed no SAI. The infection status of 32 (19%) patients was classified as indeterminate. Of the 74 patients in cohort 2, 33 (45%) achieved a favorable functional outcome after rehabilitation, while in 40 (54%) functional outcome was unfavorable. For one patient, the documentation from the rehabilitation center only provided insufficient data, this patient was therefore excluded from mRS related analyses.

### Analysis of NET markers in stroke thrombi and blood

Of the 166 patients included in this study, we were able to retrieve and analyze 106 thrombi for comparison with blood derived NET markers. Both DNA-histone 1 complexes and MPO could be found in the analyzed thrombi. The raw integrated density of the fluorescent signal relative to that of the counterstaining were 65.24% (DNA-histone 1 complexes) and 49.64% (MPO) respectively (Fig. 1). The raw values of the analyses, including additional percentile data, can be found in supplementary table ST 1.

Blood derived markers were measured at both onset and after 7 days in all patients. At onset the median concentration of cfDNA in blood was 0.19 µg/ml increasing to 0.30 µg/ml at 7 days. Median DNase activity at onset was 4.33 pmol/min/ml increasing to 4.96 pmol/min/ml at 7 days. The measurement of MPO-histone complexes resulted in a median 0.42 AU at onset increasing to 0.49 AU at 7 days.

As shown in Table 2, a difference in the median values of cfDNA at baseline were found between cohort 1 (venous puncture) with a median of 0.21 µg/ml in cohort 1 while the median of cohort 2 (arterial puncture) was 0.15 µg/l (*p* < 0.001). Median DNase activity was also different (cohort 1: 3.62 pmol/min/ml; cohort 2: 6.89 pmol/min/ml; *p* < 0.001). MPO-histone complexes showed no significant difference (0.43 AU vs. 0.43 AU; *p* = 0.63).

**Table 2:** Median of raw values for NET markers in blood at baseline divided by cohorts. Blood in cohort 1 was taken via venous puncture, while samples for cohort 2 were taken via the arterial groin puncture used for thrombectomy

Marker	Unit	Cohort 1 (venous puncture)	Cohort 2 (arterial puncture)	*P*-value
cfDNA	µg/ml [Q1-Q3]	0.21 [0.17–0.28]	0.15 [0.12–0.27]	< 0.01
DNase activity	pmol/min/ml [Q1-Q3]	3.62 [3.10–4.21]	6.89 [5.31–7.90]	< 0.01
MPO-histone complexes	AU [Q1-Q3]	0.43 [0.33–0.77]	0.43 [0.25–0.85]	0.63

Q = quartile

The differences in median value between cohorts persisted at 7 days for both cfDNA (0.39 µg/ml [cohort 1] vs. 0.20 µg/ml [cohort 2]; *p* < 0.01) and DNAse activity (4.49 pmol/min/ml [cohort 1] vs. 6.28 [cohort 2]; *p* < 0.01). However, when adjusting for age, the difference in cfDNA was found to be confounded (*p* = 0.22). MPO-Histone complexes remained without difference (0.49 AU [cohort 1] vs. 0.49 [cohort 2]; *p* = 0.86). An overview of the raw comparison can be found in supplementary table ST2.

Within thrombi DNA-histone-1 complexes and MPO correlated with each other (*ρ* = 0.792; *p* < 0.001). When comparing thrombus and blood markers, MPO in the thrombi decreased with increasing density of MPO-histone complexes in blood, showing a significant inverse correlation at baseline (*ρ* = -0.237; *p* = 0.017) and seven days (*ρ* = -0.231; *p* = 0.045). No correlation could be found between MPO in thrombi and DNase activity in the blood samples neither at baseline (*ρ* = 0.147; *p* = 0.136) nor at seven days (*ρ* = 0.072; *p* = 0.520). Also, no correlation was found between MPO in thrombi and cfDNA (baseline: *ρ* = 0.103; *p* = 0.295; seven days: *ρ* = -0.023; *p* = 0.840).

Comparing DNA-histone-1 complexes in thrombi and blood samples, a higher density of DNA-histone-1 complexes correlated with higher DNase activity at baseline (*ρ* = 0.204; *p* = 0.037). However, this correlation was weakened at day 7 (*ρ* = 0.193, *p* = 0.085). A correlation between DNA-histone-1 complexes in thrombi and cfDNA, as well as with MPO-histone complexes, in blood could not be shown (cfDNA baseline: *ρ* = 0.066; *p* = 0.504; seven days: *ρ* = 0.048; *p* = 0.671; MPO-histone: baseline: *ρ* = -0.137; *p* = 0.171; seven days: *ρ* = -0.208; *p* = 0.071).

**Fig. 2:**
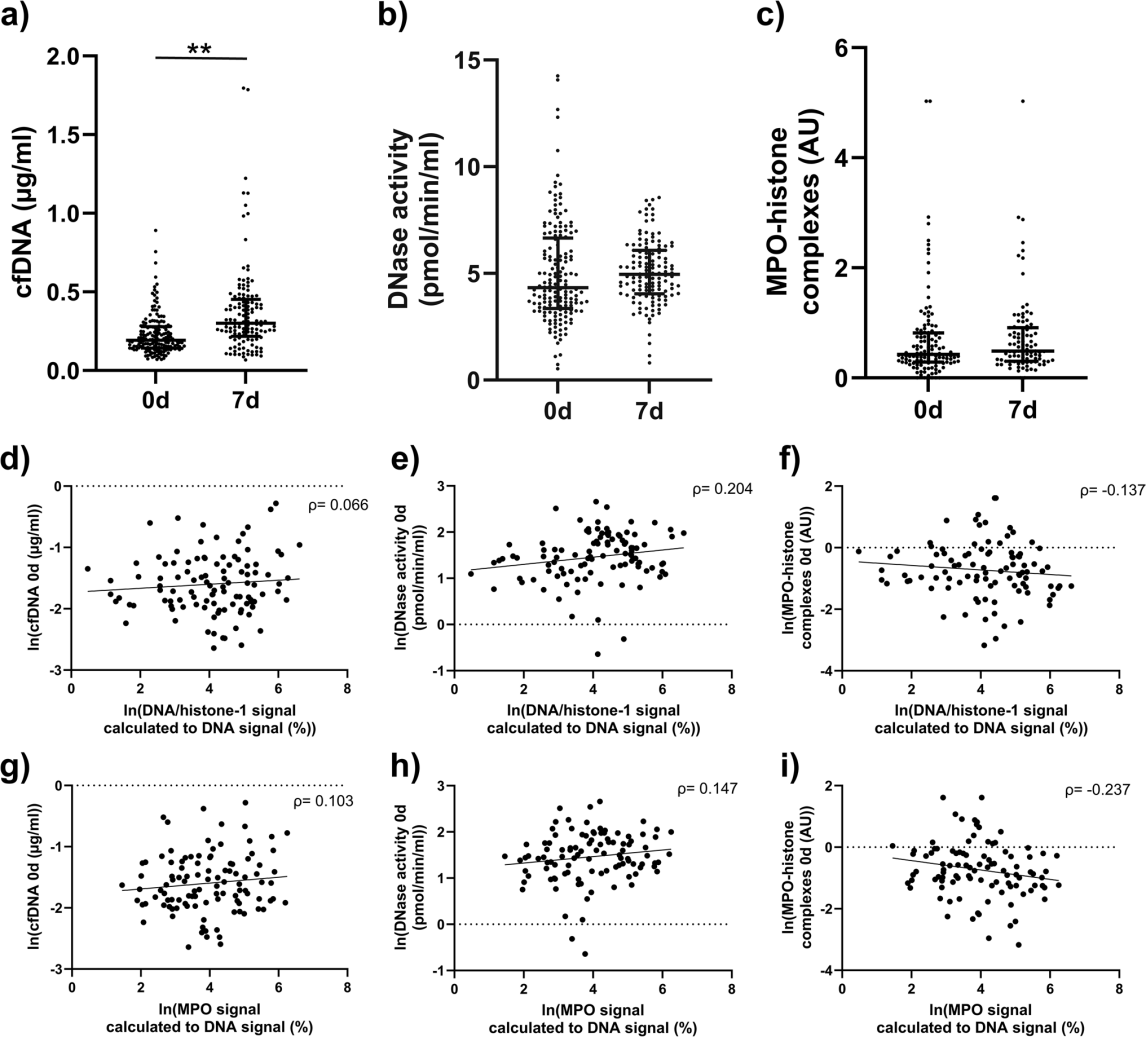
**a-c** Scatterplots of raw values for the analyzed NET markers in blood. The bars show the median and interquartile range (**p < 0.01. **d-i** Graphical representations of correlations between thrombus and blood derived NET-Markers represented as scatterplots. The correlation coefficient given is Spearman’s ρ. Visualization of a linear regression is also provided. All values are given as natural logarithm. Part **d**-**f** show comparisons of MPO in thrombi with the three blood markers (MPO-histone complexes, DNase activity and cfDNA). Part **g**-**i** show the comparison of thrombus MPO with above mentioned three blood markers. While the other correlations are positive the correlations of blood derived MPO-histone complexes with both thrombus markers are negative. The figure shows the data at baseline, additional figures for blood samples at 7 days can be found in the supplemental material (supplementary figure SFig. 2). This figure was created with GraphPad Prism, Adobe Illustrator, Adobe Photoshop and Microsoft Powerpoint

As for comparing the different blood derived NET markers with each other, only a weak negative correlation for cfDNA and DNase activity at baseline could be found (*ρ* = -0.175, *p* = 0.025). This correlation was stronger at seven days (*ρ* = -0.351; *p* < 0.001). For a graphical representation of correlation data see Fig. 2. For an overview of all correlations see supplementary figure SFig. 3.

### Comparison of thrombus and blood derived NET markers with clinical outcomes and etiology

To evaluate the impact of NET markers on patients’ clinical outcomes we grouped the patients according to mTICI-score, occurrence of SAI and mRS after rehabilitation.

Patients with favorable reperfusion (mTICI > 2b in anterior circulation or mTICI > 2a in posterior circulation) showed higher concentrations of MPO-histone complexes in baseline blood samples (0.372 vs 0.482 AU; *p* = 0.028). However, when adjusting for clinical parameters (intravenous thrombolysis and stroke etiology) this association could be shown to be confounded (OR for favorable reperfusion per MPO-histone AU increase = 1.231 [0.778–1.950] crude OR = 1.159 [0.787–2.014]).). No difference of MPO-histone complexes between patients with favorable vs. unfavorable reperfusion was found at seven days (0.483 vs 0.567 AU; *p* = 0.594).

When evaluating the NET-marker differences according to mRS after rehabilitation, etiology and SAI no significant differences could be detected. All results are summarized in supplementary tables ST3-8.

### Cytokine analysis

**Fig. 3:**
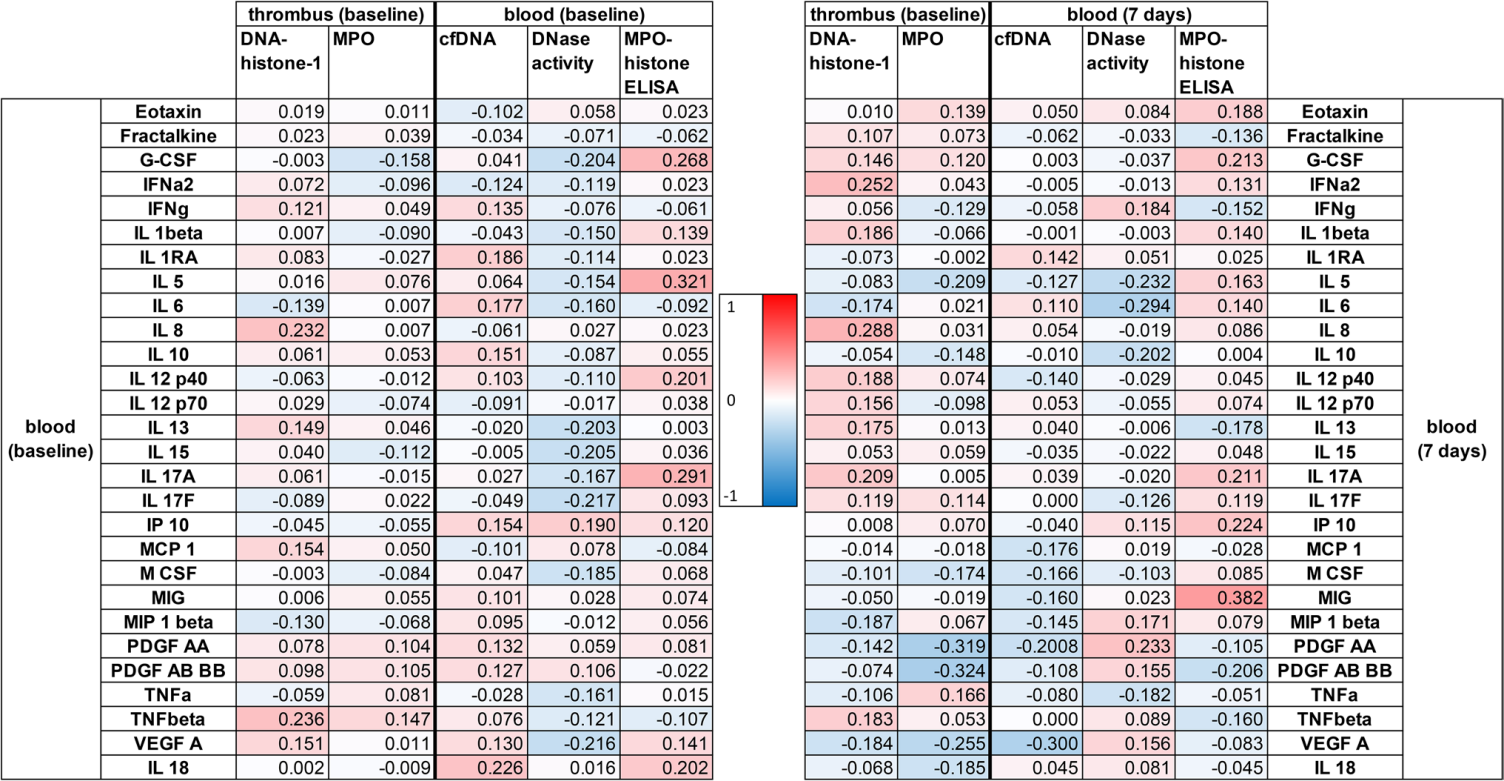
Heatmap of correlations (Spearman’s ρ) between NET markers in stroke thrombi and blood samples with cytokine profiles. The heatmap is divided in two parts based on the time of the blood sampling. The left side are measurements from blood taken at baseline. The right section shows the correlations with blood samples taken at seven days. The colors are red for positive correlations, blue for negative correlations and white for no correlation. The color intensity represents the strength of the correlation This Figure was created with Microsoft Excel and Adobe Illustrator

Cytokines were measured in cohort 2 (n = 74). An overview of correlations between NET markers and cytokines can be seen in Fig. 3. The correlations between individual cytokines are shown in supplementary figure SFig. 4

A positive correlation between MPO-histone complexes and interleukin (IL)-5 was detected at baseline, but not after seven days (baseline: *ρ* = 0.321, *p* = 0.005; seven days: *ρ* = 0.163, *p* = 0.249). Moreover, MPO-histone complexes correlated with monokine induced by gamma-interferon (MIG) at seven days, but not at baseline (seven days: *ρ* = 0.382, *p* = 0.005; baseline: *ρ* = 0.074, *p* = 0.531). cfDNA correlated with vascular endothelial growth factor A (VEGF-A) at seven days but not at baseline (seven days: *ρ* = -0.300, *p* = 0.031; baseline: *ρ* = 0.130, *p* = 0.286). Regarding NET markers in thrombi, only MPO inversely correlated with platelet derived growth factor (PDGF) at seven days, but not at baseline (PDGF AA: seven days: *ρ* = -0.319, *p* = 0.062, baseline *ρ* = 0.104, *p* = 0.466 and PDGF AB/BB: seven days: *ρ* = -0.324 *p* = 0.058, baseline: *ρ* = 0.105 *p* = 0.463).

## Discussion

In recent years, NETs have been discussed as an additional factor in the pathogenesis of stroke [3, 7, 23]. Our study, once again, confirms the presence of NETs in stroke thrombi. As NETs are already known to promote blood clotting through various pathways [5], their involvement in the formation of arterial thrombi seems plausible [24]. However, as NETs are present, regardless of thrombus age, it is likely, that NETs do not just promote thrombus formation, but also stabilize these thrombi and prevent their degradation [25].

When comparing the raw data of our two cohorts, it is noticeable that venous and arterial samples differed in terms of their cfDNA and DNase activity, while MPO-histone complexes showed no significant difference (Table 2). The differences in sample collection are one of the limitations of the study. However, this also shows that sample site must be considered as a relevant factor, when evaluating cfDNA and DNase in patients with ischemic stroke. The values also differed in the venous day 7 blood samples. While the difference in cfDNA was found to be confounded a difference in DNase activity remains. However, this could also be a result of the fact that due to the different timeframes of sample acquisition, both cohorts were measured at different points in time, which might have influenced this specific assay. Normal values for the measured NET markers are not yet available. In one study NET markers were determined in venous blood within 72 h of the acute event. The values detected for cfDNA were significantly higher than the values we measured at baseline (0.428 µg/ml vs 0.190 µg/ml) and even higher than those we measured at 7 days 0.3 µg/ml [10] However, we did show that cfDNA concentration changes over time and these samples were taken later than our onset samples. Data from the hyperacute phase of stroke are rare. In one study, NET markers were also measured in arterial samples during thrombectomy and the values for cfDNA were consistent with ours [26]

To our knowledge, our group is the first to study endogenous DNase activity in patients after ischemic stroke, so there is no comparative data available. With an increasing number of studies focusing on the therapeutic use of DNase in the treatment of NET-associated diseases, further insight into the enodogenic regulatory mechanisms of NET- and consecutive thrombus formation seems imperative.

A major focus of our study was to evaluate whether the different blood derived NET markers actually correlate with the amount of NETs in cerebral thrombi in the hyper-acute setting of stroke. As shown in Fig. 2 i concentrations of MPO-histone complexes in blood were inversely correlated with the amount of MPO in thrombi, even if the correlation was rather weak and has to be confirmed in larger samples. Based on the aforementioned regulatory role of endogenous DNase activity we also analyzed the correlation between DNase activity in the hyperacute phase of stroke and NETs content in cerebral thrombi and found higher DNase activity correlated significantly with higher amounts of DNA-histone-1 complexes in thrombi (Fig. 2 e), although the effect size was low (*ρ* = 0.204; *p* = 0.037) Therefore, studies with more patients are necessary in order to better record and understand correlations. As DNase is capable of NET degradation [27], one might assume that DNase activity is enhanced in order to regulate NET formation with higher amount of NETs leading to a higher release of NET degrading enzymes like DNase.

This may also indicate the potential of DNase in the treatment of acute ischemic stroke, as other studies have shown that DNase can improve the lysis of thrombi resistant to recombinant tissue plasminogen activator (rt-PA) [6, 28, 29]. Moreover, studies in mice showed that the application of DNase may also reduce the brain damage resulting from ischemic stroke [28, 30] and furthermore the subsequent risk for recurrent vascular events [31]. The application in humans is also currently tested by Campbell et al. in a clinical phase 2 trial (ClinicalTrials.gov Identifier: NCT05203224).

For cfDNA however, we were not able to show any correlation with the amount of NETs in thrombi (Fig. 2 d and g). This might be explained by the fact that cfDNA is not specific for NET-formation but rather a general marker of cell death, as it is elevated in patients with more severe strokes [10]. cfDNA is also a mediator of inflammation [32]. Presumably the effect of brain damage incurred by ischemic stroke and the subsequent inflammatory reaction are large enough to mask thrombus-related cfDNA alterations.

A prior study suggested an association between thrombus DNA and stroke etiology [8]. However, we were not able to find similar results for DNA-histone-1 complexes or MPO (supplementary Table ST5) This could be attributed to differences in study design, as our study was primarily designed to more specifically measure the correlation of NETs in thrombi with blood markers in general. The number of patients might therefore not have been sufficient to detect smaller differences based on the multiple different etiologies.

As for the analyzed cytokines at baseline (Fig. 3), we only detected a notable correlation between MPO-histone complexes and IL-5. IL-5 has been discussed as a potential predictive factor for positive functional outcome after stroke [33]. Interestingly, our results indicated a similar, although confounded, association between MPO-histone complexes and recanalization, as the amount of MPO-histone complexes was higher in patients with favorable recanalization outcome. However, further studies would be necessary to confirm its predictive value. We also found several cytokines that were associated with NET markers after seven days (MIG, VEGF-A, PDGF-AA and PDGF-AB/BB). However, as none of these showed any notable correlations at baseline, a connection between those cytokines and the role of NETs in stroke formation seems unlikely. One might assume that other contributing factors in the hyper-acute phase of acute ischemic stroke bias the influences of NETs on cytokine reactions.

Apart from the aforementioned association between MPO-histone complexes and recanalization, we were unable to show any significant association between NETs in thrombi, as well as NET markers in blood, with SAI, stroke etiology or functional outcome. However, as earlier studies were able to find some associations between blood derived NET markers and SAI [13], etiology and mortality [10], larger studies might still uncover further predictive and diagnostic uses of those markers.

## Conclusions

In conclusion, this is the first study investigating the association of regulatory mechanisms of NETs and their content in cerebral thrombi. Although the detected correlations are weak, our results provide a starting point for further investigations of the potentially crucial targets of NETs regulation in stroke.

NET markers in the thrombus showed good concordance with each other. Our results also suggest an association between better recanalization outcomes and higher levels of MPO-histone complexes. However, further studies are needed to confirm this and to find other associations between NETs and clinical outcomes.

### Supplementary Information

Below is the link to the electronic supplementary material.

Supplementary file1 (PDF 11.2 MB)

## Data Availability

The data that support the findings of this study are available, but restrictions apply to the availability of these data, which were used under licence for the current study and so are not publicly available. The data are, however, available from the authors.

## Declarations

All patients or, if they were unable to, their proxies provided written informed consent to participation in this study.
