# Supplementary material for: Assessment of associations between neutrophil extracellular trap biomarkers in blood and thrombi in acute ischemic stroke patients
Source: J Thromb Thrombolysis. 2024 Jun 9;57(6):936–46. doi: 10.1007/s11239-024-03004-y (PMC11315804; doi:10.1007/s11239-024-03004-y)
Supplement: Supplementary file 1 — Supplementary file1 (PDF 11.2 MB) [file 11239_2024_3004_MOESM1_ESM.pdf]

SUPPLEMENTAL MATERIAL

Assessment of associations between neutrophil extracellular trap biomarkers in blood and thrombi in acute ischemic stroke patients

Tristan Baumann[1*], Nicole de Buhr, PhD[2,3*], Nicole Blume[1], Maria M. Gabriel, MD [1], Johanna Ernst, MD [1], Leonie Fingerhut, PhD[2,3], Rabea Imker[2,3], Omar Abu-Fares, MD[4], Mark Kühnel, PhD[5,6], Prof. Danny D. Jonigk, MD[6,7], Friedrich Götz, MD[4], Prof. Christine Falk, PhD[8], Prof. Karin Weissenborn, MD[1], Gerrit M. Grosse, MD[1,9*], Ramona Schuppner, MD[1*]

[1] Department of Neurology, Hannover Medical School, Hannover, Germany

[2] Department of Biochemistry, University of Veterinary Medicine Hannover, Hannover, Germany

[3] Research Center for Emerging Infections and Zoonoses (RIZ), University of Veterinary Medicine Hannover, Hannover, Germany

[4] Institute of Diagnostic and Interventional Neuroradiology, Hannover Medical School, Hannover, Germany

[5] Institute of Pathology, Hannover Medical School, Hannover, Germany

[6] Member of the German Center for Lung Research (DZL), Biomedical Research in Endstage and Obstructive Lung Disease Hannover (BREATH), Hannover, Germany

[7] Institute of Pathology, RWTH Aachen Medical University, Aachen, Germany

[8]Institute of Transplant Immunology, Hannover Medical School, Hannover, Germany

[9]Department of Neurology and Stroke Center, University Hospital Basel, Basel, Switzerland

* Contributed equally

Correspondence:

Ramona Schuppner, MD

Department of Neurology

Hannover Medical School

Carl-Neuberg-Str. 1

30625 Hannover, Germany

Phone: +49-511-532-3580

e-mail: schuppner.ramona@mh-hannover.de

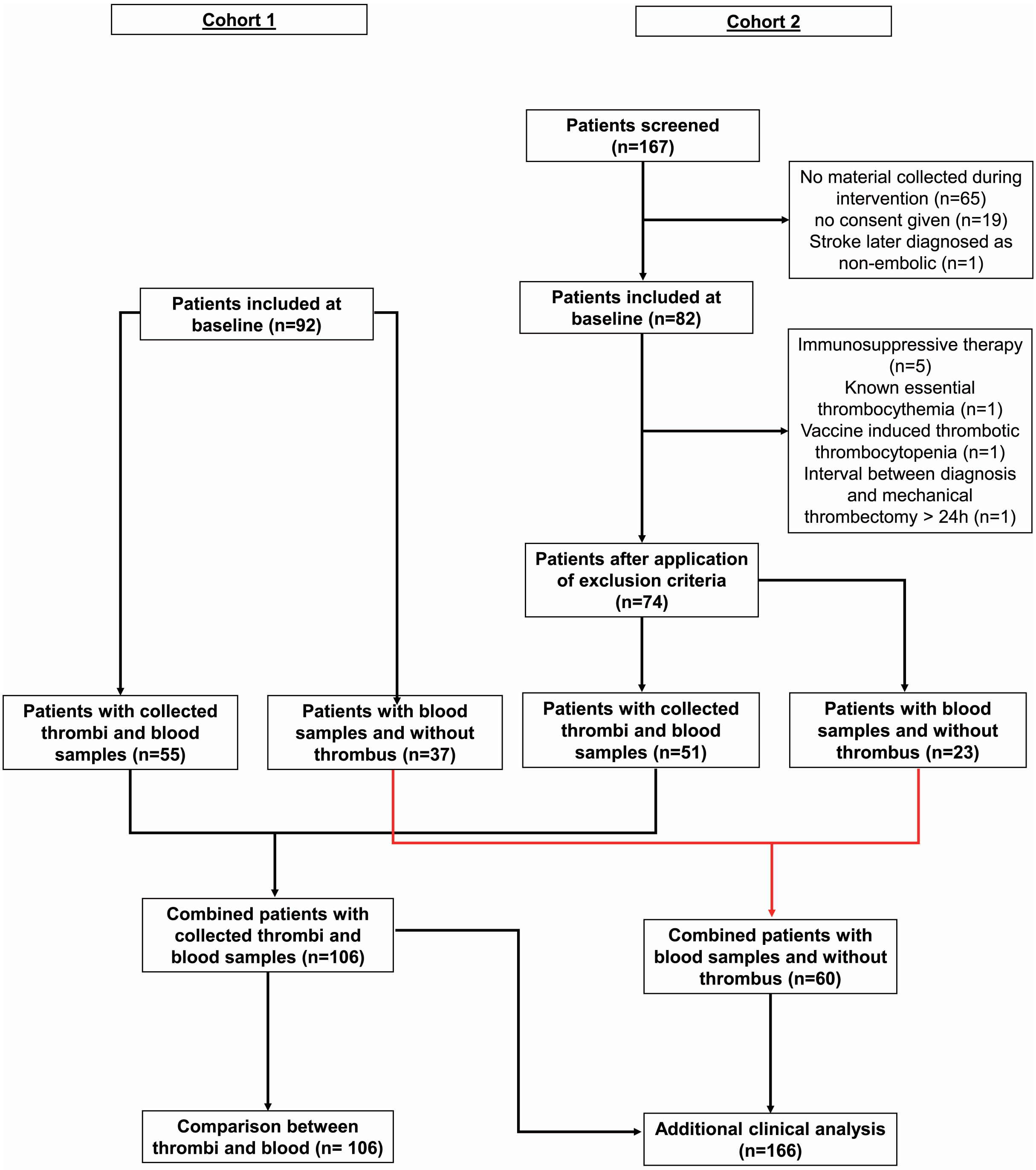

Supplemental Figure SFig. 1: Flowchart of patient recruitment and available thrombi for analysis

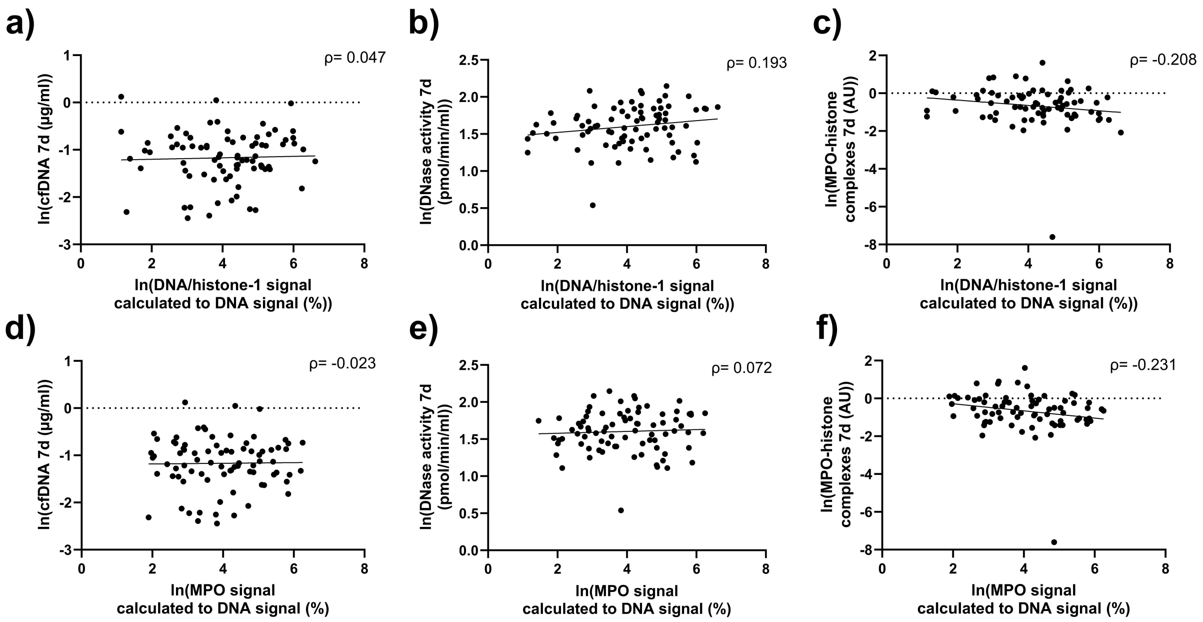

Supplemental Figure SFig. 2 Graphical representations of correlations between thrombus and blood derived NET-Markers at 7 days represented as scatterplots. The correlation coefficient given is Spearmans' ρ. Visualization of a linear regression is also provided. All values are given as natural logarithm. Part a)-c) show comparisons of MPO in thrombi with the three blood markers (MPO-Histone complexes, DNase activity and cfDNA). Part d)-f) show the comparison of thrombus MPO with above mentioned three blood markers.

	DNA-Histone-1	MPO	cfDNA0d	cfDNA 7d	DNase activity 0d	DNase activity 7d	MPO-histone ELISA 0d	MPO-histone ELISA 7d
**DNA-Histone-1**	1.000	0.792	0.066	0.047	0.204	0.193	-0.137	-0.208
**MPO**		1.000	0.103	-0.023	0.147	0.072	-0.237	-0.231
**cfDNA0d**			1.000	0.483	-0.175	-0.233	0.026	0.100
**cfDNA 7d**				1.000	-0.389	-0.351	0.114	0.092
**DNase activity 0d**					1.000	0.611	-0.094	-0.115
**DNase activity 7d**						1.000	-0.060	-0.113
**MPO-histone ELISA 0d**							1.000	0.583
**MPO-histone ELISA 7d**								1.000

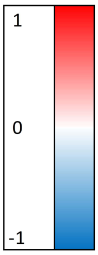

Supplementary Figure SFig. 3: Heatmap of correlations (Spearman’s ρ) between the individual thrombus and blood derived NET markers at baseline and after seven days. The heatmap is divided by thrombus and blood derived NET markers. The colors are red for positive correlations, blue for negative correlations and white for no correlation. The color intensity represents the strength of the correlation.

a)

	Eotaxin	Fractalkine	G CSF	IFN α2	IFN γ	IL 1β	IL 1RA	IL 5	IL 6	IL 8	IL 10	IL 12 p40	IL 12 p70	IL 13	IL 15	IL 17A	IL 17F	IL10	MCP 1	M CSF	MIG	MIP 1β	PDGF AA	PDGF AB BB	TNFα	TNFβ	VEGF A	IL 18
**Eotaxin**	1.000	0.072	0.119	0.098	-0.051	0.115	0.145	0.103	-0.054	0.313	-0.030	0.108	0.136	-0.012	0.127	0.166	0.046	0.174	0.437	0.258	0.069	0.320	0.127	0.196	0.115	-0.037	0.176	0.295
**Fractalkine**		1.000	0.380	0.570	0.574	0.412	0.409	0.471	0.041	0.338	0.047	0.353	0.503	0.810	0.500	0.430	0.450	0.131	0.292	0.457	0.262	0.376	0.006	-0.058	0.718	0.708	0.201	0.010
**G CSF**			1.000	0.339	0.303	0.284	0.225	0.300	0.331	0.436	0.170	0.330	0.319	0.357	0.415	0.376	0.334	0.221	0.291	0.388	0.233	0.263	-0.025	-0.146	0.399	0.261	0.356	0.091
**IFN α2**				1.000	0.290	0.607	0.380	0.261	0.117	0.337	0.108	0.151	0.310	0.451	0.533	0.685	0.392	0.054	0.053	0.331	0.007	0.237	-0.022	-0.067	0.306	0.377	0.262	-0.146
**IFN γ**					1.000	0.411	0.423	0.281	0.118	0.206	0.184	0.129	0.257	0.592	0.431	0.379	0.306	0.069	0.264	0.466	0.253	0.314	-0.166	-0.175	0.487	0.623	0.243	0.182
**IL 1β**						1.000	0.516	0.328	0.148	0.409	0.219	0.162	0.206	0.340	0.659	0.756	0.439	0.049	0.141	0.388	0.048	0.381	0.137	0.076	0.330	0.341	0.299	0.089
**IL 1RA**							1.000	0.225	0.240	0.245	0.254	0.269	0.191	0.366	0.430	0.421	0.397	0.142	0.179	0.411	0.222	0.243	0.056	0.032	0.497	0.456	0.223	0.178
**IL 5**								1.000	0.078	0.368	0.081	0.421	0.267	0.367	0.511	0.391	0.264	0.167	0.287	0.376	0.338	0.367	-0.150	-0.176	0.449	0.253	0.114	0.253
**IL 6**									1.000	0.303	0.478	0.141	0.003	-0.004	0.235	0.045	0.143	0.142	0.239	0.269	0.140	0.142	-0.073	-0.038	0.159	0.026	0.131	0.130
**IL 8**										1.000	0.215	0.342	0.220	0.098	0.528	0.228	0.274	0.275	0.444	0.368	0.170	0.344	0.111	0.081	0.226	0.154	0.278	0.226
**IL 10**											1.000	0.244	0.172	0.181	0.102	0.235	0.187	0.077	0.151	0.148	0.234	0.229	-0.080	-0.054	0.159	0.192	0.198	0.133
**IL 12 p40**												1.000	0.355	0.221	0.129	0.086	0.283	0.489	0.255	0.340	0.511	0.368	-0.120	-0.106	0.440	0.126	0.218	0.250
**IL 12 p70**													1.000	0.555	0.244	0.277	0.381	0.176	0.334	0.321	0.218	0.342	-0.089	-0.096	0.501	0.445	0.224	0.043
**IL 13**														1.000	0.348	0.499	0.420	-0.033	0.197	0.354	0.118	0.318	-0.026	-0.081	0.685	0.780	0.346	-0.093
**IL_15**															1.000	0.520	0.452	0.042	0.241	0.497	0.094	0.257	-0.116	-0.125	0.363	0.319	0.179	0.217
**IL 17A**																1.000	0.403	0.106	0.045	0.268	0.044	0.359	0.105	-0.015	0.335	0.387	0.354	-0.040
**IL 17F**																	1.000	0.044	0.064	0.357	0.217	0.338	-0.057	-0.117	0.474	0.298	0.162	0.021
**IL 10**																		1.000	0.207	0.270	0.511	0.348	0.072	0.078	0.226	-0.096	0.219	0.215
**MCP 1**																			1.000	0.364	0.269	0.392	0.054	0.090	0.376	0.287	0.223	0.367
**M CSF**																				1.000	0.296	0.402	-0.207	-0.222	0.457	0.332	0.237	0.407
**MIG**																					1.000	0.278	-0.099	-0.165	0.406	0.076	0.056	0.278
**MIP 1β**																						1.000	0.128	0.107	0.386	0.329	0.444	0.298
**PDGF AA**																							1.000	0.909	0.028	-0.035	0.391	0.054
**PDGF AB BB**																								1.000	-0.034	-0.136	0.387	0.081
**TNF α**																									1.000	0.615	0.278	0.163
**TNF β**																										1.000	0.276	0.038
**VEGF A**																											1.000	0.132
**IL 18**																												1.000

b)

	Eotaxin	Fractalkine	G CSF	IFN α2	IFN γ	IL 1β	IL 1RA	IL 5	IL 6	IL 8	IL 10	IL 12 p40	IL 12 p70	IL 13	IL 15	IL 17A	IL 17F	IL10	MCP 1	M CSF	MIG	MIP 1β	PDGF AA	PDGF AB BB	TNFα	TNFβ	VEGF A	IL 18
**Eotaxin**	1.000	0.377	0.144	0.400	0.290	0.271	0.087	0.052	-0.084	0.381	0.189	0.178	0.367	0.343	0.355	0.390	0.439	0.312	0.298	0.218	0.202	0.568	-0.033	0.133	0.383	0.343	0.238	0.005
**Fractalkine**		1.000	0.437	0.652	0.609	0.356	0.266	0.211	0.094	0.261	0.477	0.555	0.670	0.889	0.496	0.518	0.721	0.208	0.312	0.602	0.213	0.394	-0.161	-0.068	0.768	0.815	0.137	-0.108
**G CSF**			1.000	0.618	0.359	0.515	0.354	0.384	0.260	0.319	0.525	0.449	0.510	0.431	0.440	0.626	0.577	0.250	0.129	0.240	0.257	0.306	-0.221	-0.191	0.410	0.381	0.310	0.057
**IFN α2**				1.000	0.514	0.670	0.301	0.476	0.259	0.501	0.655	0.539	0.775	0.632	0.694	0.812	0.786	0.198	0.172	0.459	0.253	0.451	-0.146	-0.106	0.563	0.550	0.286	-0.061
**IFN γ**					1.000	0.466	0.259	0.217	0.032	0.191	0.491	0.314	0.548	0.661	0.429	0.525	0.392	0.249	0.159	0.302	0.286	0.250	-0.055	-0.071	0.482	0.704	0.258	-0.094
**IL 1β**						1.000	0.314	0.473	0.125	0.464	0.513	0.295	0.488	0.440	0.628	0.858	0.553	0.173	0.097	0.222	0.334	0.340	0.032	0.007	0.465	0.378	0.485	-0.028
**IL 1RA**							1.000	0.254	0.485	0.242	0.201	0.328	0.255	0.212	0.476	0.268	0.364	0.055	0.034	0.235	0.138	0.023	-0.273	-0.171	0.391	0.297	0.107	0.134
**IL 5**								1.000	0.278	0.446	0.487	0.251	0.249	0.314	0.555	0.462	0.225	-0.086	0.004	0.333	0.133	0.154	-0.030	-0.065	0.303	0.180	0.280	0.112
**IL 6**									1.000	0.321	0.263	0.118	0.221	0.054	0.409	0.162	0.154	0.016	-0.043	0.110	0.054	0.017	-0.210	-0.159	0.166	0.116	-0.014	-0.011
**IL 8**										1.000	0.504	0.308	0.414	0.295	0.593	0.466	0.363	0.453	0.285	0.254	0.265	0.515	-0.096	0.015	0.388	0.247	0.316	0.136
**IL 10**											1.000	0.494	0.607	0.531	0.490	0.618	0.583	0.259	0.251	0.471	0.323	0.426	-0.404	-0.349	0.521	0.384	0.294	0.074
**IL 12 p40**												1.000	0.455	0.460	0.355	0.344	0.548	0.303	0.384	0.560	0.325	0.368	-0.406	-0.356	0.581	0.438	0.089	0.210
**IL 12 p70**													1.000	0.658	0.488	0.665	0.648	0.259	0.166	0.427	0.305	0.397	-0.258	-0.158	0.611	0.598	0.109	-0.212
**IL 13**														1.000	0.507	0.572	0.652	0.176	0.280	0.483	0.185	0.334	-0.140	-0.079	0.689	0.853	0.236	-0.117
**IL_15**															1.000	0.628	0.563	0.201	0.194	0.483	0.268	0.392	-0.203	-0.118	0.564	0.418	0.418	0.057
**IL 17A**																1.000	0.662	0.229	0.048	0.287	0.371	0.398	-0.048	-0.105	0.478	0.461	0.388	-0.083
**IL 17F**																	1.000	0.278	0.358	0.466	0.242	0.382	-0.312	-0.208	0.640	0.530	0.191	-0.117
**IL 10**																		1.000	0.530	0.221	0.508	0.505	-0.183	-0.180	0.263	0.189	0.205	0.131
**MCP 1**																			1.000	0.305	0.329	0.387	-0.322	-0.131	0.460	0.288	0.081	0.199
**M CSF**																				1.000	0.259	0.372	-0.212	-0.116	0.518	0.346	0.197	0.158
**MIG**																					1.000	0.277	-0.313	-0.424	0.395	0.156	0.125	0.022
**MIP 1β**																						1.000	-0.003	0.054	0.494	0.353	0.415	0.247
**PDGF AA**																							1.000	0.810	-0.378	-0.117	0.305	0.015
**PDGF AB BB**																								1.000	-0.244	-0.092	0.243	-0.004
**TNF α**																									1.000	0.646	0.167	0.002
**TNF β**																										1.000	0.175	-0.108
**VEGF A**																											1.000	0.318
**IL 18**																												1.000

Supplemental Figure SFig. 4: Heatmaps for the correlation (Spearman's rho) of the analyzed cytokines a) for onset (0d) and b) the 7 day follow-up (7d), respectively. The colors are red for positive correlations, blue for negative correlations and white for no correlation. The color intensity represents the strength of the correlation.

	Marker	Unit	onset (0d)	7 days
**Thrombus derived**	**DNA-histone 1 complexes**	Units	65.24 [22.99-162.18]	not applicable
	**MPO**	Units	49.65 [20.39-128.67]	not applicable
**Blood derived**	**cfDNA**	ng/ml	0.19 [0.14-0.28]	0.3 [0.22-0.45]
	**DNase activity**	pmol/min/ml	4.33 [3.36-6.66]	4.96 [4.05-6.08]
	**MPO-histone Complexes**	AU	0.42 [0.29-0.82]	0.49 [0.3-0.91]

Supplementary Table ST1: Raw values for the analyzed NET markers. The table shows the median values as well as the values for the 25th and 75th percentile.

			mTICI		
			<2c (<2b posterior circulation)	≥2c (2b posterior circulation)	p-value
Thrombus derived	DNA-histone 1 complexes	Units	63.02 (30.45-159.17)	67.77 (18.99-162.45)	0.70
	MPO	Units	48.32 (18.48-126.79)	49.65 (22.05-129.49)	0.86
Blood derived	cfDNA (onset)	ng/ml	0.18 (0.14-0.28)	0.20 (0.15-0.28)	0.30
	cfDNA (7d)	ng/ml	0.31 (0.22-0.47)	0.30 (0.2-0.43)	0.42
	DNase activity (onset)	pmol/min/ml	4.50 (3.49-6.91)	4.17 (3.25-5.89)	0.33
	DNase activity (7d)	pmol/min/ml	5.25 (4.1-6.26)	4.79 (4-6.03)	0.65
	MPO-histone complexes (onset)	AU	0.37 (0.23-0.72)	0.48 (0.34-0.83)	0.03
	MPO-histone complexes (7d)	AU	0.48 (0.29-0.92)	0.57 (0.3-0.89)	0.82

Supplementary Table ST2: Mann-Whitney-U Test for differences between NET markers for different recanalization outcomes according to mTICI (Modified treatment in cerebral infarction) score

			stroke acquired infections			
			no	yes	indeterminate	p-value
Thrombus derived	DNA-histone 1 complexes	Units	81.12 (23.79-177.47)	45.57 (19.55-108.23)	77.44 (61.49-165.45)	0.10
	MPO	Units	50.2 (18.31-150.52)	56.26 (17.78-128.53)	45.07 (29.19-85.47)	0.96
Blood derived	cfDNA (onset)	ng/ml	0.19 (0.14-0.25)	0.20 (0.14-0.31)	0.19 (0.14-0.28)	0.49
	cfDNA (7d)	ng/ml	0.30 (0.24-0.43)	0.35 (0.20-0.54)	0.25 (0.12-0.39)	0.06
	DNase activity (onset)	pmol/min/ml	4.19 (3.49-5.64)	4.40 (3.12-6.98)	5.11 (2.92-7.22)	0.60
	DNase activity (7d)	pmol/min/ml	4.95 (4.23-5.94)	4.63 (3.51-5.94)	5.76 (4.65-7.48)	<0.01
	MPO-histone complexes (onset)	AU	0.39 (0.27-0.81)	0.45 (0.29-0.94)	0.45 (0.3-0.76)	0.77
	MPO-histone complexes (7d)	AU	0.47 (0.3-0.91)	0.48 (0.3-0.94)	0.63 (0.31-0.8)	0.99

Supplementary Table ST3: Kruskal-Wallis-H Test for differences in NET markers based on stroke acquired infections

			TOAST				
			Large artery athero-sclerosis	Cardio-embolic	Crypto-genic	Dissection	p-value
Thrombus derived	DNA-histone 1 complexes	Units	126.45 (38.26-167.92)	62.94 (22-138.06)	71.65 (22.59-140.67)	4.02 (4.02-4.02)	0.26
	MPO	Units	87.72 (24.79-152.65)	49.11 (18.35-129.88)	48.93 (24.4-75.86)	11.23 (11.23-11.23)	0.44
Blood derived	cfDNA (onset)	ng/ml	0.19 (0.15-0.26)	0.18 (0.14-0.29)	0.20 (0.15-0.25)	0.17 (0.16-0.21)	0.99
	cfDNA (7d)	ng/ml	0.26 (0.22-0.39)	0.29 (0.20-0.46)	0.34 (0.26-0.51)	0.30 (0.30-0.31)	0.60
	DNase activity (onset)	pmol/min /ml	5.27 (4.14-8.45)	4.26 (3.21-6.47)	3.95 (3.36-6.08)	4.14 (3.24-0)	0.23
	DNase activity (7d)	pmol/min /ml	5.64 (4.45-6.47)	4.96 (4.00-6.08)	4.61 (3.61-5.75)	4.99 (4.39-0)	0.33
	MPO-histone complexes (onset)	AU	0.40 (0.26-0.76)	0.47 (0.29-0.81)	0.39 (0.25-0.99)	0.75 (0.75-0.75)	0.64
	MPO-histone complexes (7d)	AU	0.32 (0.24-0.97)	0.58 (0.36-0.87)	0.42 (0.3-1.12)	1.05 (1.05-1.05)	0.37

Supplementary Table ST4: Kruskal-Wallis-H Test for association of stroke etiology according to TOAST classification on NET markers

			Intravenous thrombolysis		
			no	yes	p-value
Thrombus derived	DNA-histone 1 complexes	Units	35.45 (16.70-107.11)	63.31 (26.42-153.76)	0.03
	MPO	Units	50.76 (14.73-135.07)	82.05 (35.8-164.69)	0.07
Blood derived	cfDNA (onset)	ng/ml	0.19 (0.14-0.28)	0.19 (0.14-0.27)	0.88
	cfDNA (7d)	ng/ml	0.31 (0.21-0.54)	0.30 (0.23-0.42)	0.68
	DNase activity (onset)	pmol/min/ml	4.36 (3.24-6.47)	4.33 (3.38-6.75)	0.81
	DNase activity (7d)	pmol/min/ml	4.70 (4.01-5.97)	5.14 (4.17-6.23)	0.21
	MPO-histone complexes (onset)	AU	0.55 (0.34-0.89)	0.40 (0.27-0.72)	0.05
	MPO-histone complexes (7d)	AU	0.60 (0.42-0.87)	0.43 (0.29-0.95)	0.20

Supplementary Table ST5: Mann-Whitney-U Test for differences in NET markers based on the application of intravenous thrombolysis.

			sex		
			female	male	p-value
Thrombus derived	DNA-histone 1 complexes	Units	53.16 (19.32-137.94)	90.26 (43.11-165.89)	0.05
	MPO	Units	34.04 (18.1-115.86)	63.64 (31.26-130.54)	0.11
Blood derived	cfDNA (onset)	ng/ml	0.18 (0.14-0.26)	0.21 (0.15-0.28)	0.15
	cfDNA (7d)	ng/ml	0.29 (0.19-0.46)	0.31 (0.24-0.43)	0.44
	DNase activity (onset)	pmol/min/ml	4.54 (3.55-6.87)	4.12 (3.24-6.29)	0.20
	DNase activity (7d)	pmol/min/ml	5.28 (4.47-6.35)	4.42 (3.88-5.57)	<0.01
	MPO-histone complexes (onset)	AU	0.41 (0.29-0.8)	0.47 (0.28-0.88)	0.62
	MPO-histone complexes (7d)	AU	0.49 (0.32-0.91)	0.48 (0.29-0.91)	0.70

Supplementary Table ST6: Mann-Whitney-U Test for differences in NET markers based on sex

			mRS after rehabilitation		
			0-2	3-6	p-value
Thrombus derived	DNA-histone 1 complexes	Units	82.38 (47.98-142.81)	82.94 (60.11-169.33)	0.54
	MPO	Units	53.47 (31.47-152.65)	67 (30.35-108.75)	0.63
Blood derived	cfDNA (onset)	ng/ml	0.15 (0.11-0.29)	0.15 (0.11-0.27)	0.79
	cfDNA (7d)	ng/ml	0.17 (0.12-0.27)	0.21 (0.11-0.43)	0.49
	DNase activity (onset)	pmol/min/ml	6.77 (5.53-8.09)	6.97 (5.22-7.81)	0.93
	DNase activity (7d)	pmol/min/ml	6.08 (5.52-7.45)	6.34 (4.86-6.91)	0.92
	MPO-histone complexes (onset)	AU	0.40 (0.22-0.68)	0.48 (0.24-1.18)	0.27
	MPO-histone complexes (7d)	AU	0.40 (0.3-0.93)	0.60 (0.31-1.09)	0.33

Supplementary Table ST7: Mann-Whitney-U Test for differences in NET markers based on clinical outcome according to modified Rankin Scale (mRS)

**parameter**	**type**	all analyzed patients (n= 124)	mTICI <2c (<2b post circulation) (n=64)	≥2c (2b post circulation) (n=60)	p-value
age	median (25th - 75th percentile)	79 (67-83)	75.5 (67-83)	79 (68-83.75)	0.506
ESRS	median (25th - 75th percentile)	3 (2-5)	3 (2-4)	4 (3-5)	0.179
NIHSS	median (25th - 75th percentile)	15 (11-19.75)	15 (11-19)	16 (11.25-20.75)	0.284
Onset to groin time (in min; when applicable)	median (25th - 75th percentile)	238 (150 - 311.75)	226 (142.5-311.5)	245 (156-350)	0.434
door to groin time (in min; when applicable)	median (25th - 75th percentile)	60 (36.5-86)	60 (38-86)	61 (33.5-85)	0.657
Onset to needle time (in min; when applicable)	median (25th - 75th percentile)	96 (74-137)	95 (71.5-137.5)	102.5 (75.25-140.75)	0.763
mRS prior to stroke	median (25th - 75th percentile)	1 (0-1)	1 (0-1)	1 (0-1.75)	0.659
secondarily transferred to our hospital	n (%)	66 (53.2)	36 (56,3)	30 (50)	0.589
sex (female)	n (%)	60 (48.4)	36 (56,3)	32 (53.3)	0.369
arterial hypertension	n (%)	104 (83.9)	55 (85.9)	49 (81.7)	0.627
diabetes	n (%)	40 (32.2)	22 (34.4)	18 (30)	0.701
dyslipidemia	n (%)	50 (40.3)	22 (34,4)	21 (35)	0.275
Obesity (BMI>30)	n (%)	37 (29.8)	20 (31.3)	17 (28.3)	0.845
coronary heart disease	n (%)	22 (17.7)	9 (14.1)	13 (21.7)	0.348
previous myocardial infarction	n (%)	14 (11.3)	6 (9.4)	8 (13.3)	0.576
previous stroke or TIA	n (%)	24 (19.4)	15 (23.4)	9 (15)	0.263
atrial fibrillation	n (%)	54 (43.5)	20 (31.3)	34 (56.7)	0.006
thrombolytic treatment	n (%)	73 (58.9)	44 (68.8)	29 (48.3)	0.028
anticoagulation	n (%)	34 (27.4)	13 (20.3)	21 (35)	0.074
unclear onset	n (%)	46 (37.1)	20 (31.3)	26 (43.3)	0.195
smoker	n (%)	38 (30.6)	18 (28.1)	20 (33.3)	0.563
alcohol addiction	n (%)	8 (6.5)	4 (6.3)	4 (6.7)	0.999
**affected territory of circulation**					0.090
**anterior**	n (%)	110 (88.7)	60 (93.8)	50 (83.3)	
**posterior**	n (%)	14 (11.3)	4 (6.3)	10 (16.7)	

**TOAST**					0.014
Large artery atherosclerosis	n (%)	22 (17.7)	15 (23.4)	7 (11.7)	
cardioembolic stroke	n (%)	71 (57.3)	29 (45.3)	42 (70)	
cryptogenic stroke	n (%)	30 (24.2)	20 (31.3)	10 (16.7)	
artery dissection	n (%)	1 (0.8)	0 (0)	1 (1.7)	

Supplementary Table ST8: Identification of differences between patients grouped by recanalization outcome. ESRS= Essen stroke risk scale; NIHSS= National Institute of Health Stroke Scale; mRS= modified Rankin scale; BMI=body mass index; TIA= transient ischemic attack, TOAST= Trial of Org 10172 in Acute Stroke Treatment criteria
